# Novel gene mutations in three Japanese patients with ARC syndrome associated mild phenotypes: a case series

**DOI:** 10.1186/s13256-022-03279-w

**Published:** 2022-02-13

**Authors:** Yoshinori Satomura, Kazuhiko Bessho, Nobutoshi Nawa, Hidehito Kondo, Shogo Ito, Takao Togawa, Masanao Yano, Yuki Yamano, Taisuke Inoue, Miho Fukui, Shinsuke Onuma, Tomoya Fukuoka, Kie Yasuda, Takeshi Kimura, Makiko Tachibana, Taichi Kitaoka, Shin Nabatame, Keiichi Ozono

**Affiliations:** 1 Department of Pediatrics, Graduate School of Medicine, Osaka University, 2-2 Yamadaoka, Suita, Osaka 565-0871 Japan; 2 Department of Pediatrics and Neonatology, Nagoya City University Graduate School of Medical Sciences, Aichi, Japan; 3 Department of General Pediatrics, Osaka Women’s and Children’s Hospital, Osaka, Japan

**Keywords:** ARC syndrome, *VPS33B*, *VIPAS39*, Targeted NGS, Neonatal cholestasis, Mild phenotype, Normal GGT, Severe hemorrhage

## Abstract

**Background:**

Arthrogryposis, renal dysfunction, and cholestasis syndrome (ARCS) is a rare autosomal recessive disorder caused by mutations in *VPS33B* (ARCS1) and *VIPAS39* (ARCS2). As per literature, most patients with ARCS died of persistent infections and bleeding by the age of 1 year. We report the first Japanese cases with ARCS1 and ARCS2 who presented with mild phenotypes and were diagnosed via genetic testing.

**Case presentation:**

Case 1: A 6-year-old boy born to nonconsanguineous Japanese parents presented with jaundice and normal serum gamma-glutamyl transferase (GGT) levels, proteinuria, bilateral nerve deafness, motor delay, failure to thrive, and persistent pruritus. After cochlear implantation for deafness at the age of 2 years, despite a normal platelet count and prothrombin time-international normalized ratio, the patient presented with persistent bleeding that required hematoma removal. Although he did not show any obvious signs of arthrogryposis, he was suspected to have ARCS based on other symptoms. Compound heterozygous mutations in *VPS33B* were identified using targeted next-generation sequencing (NGS), which resulted in no protein expression. Case 2: A 7-month-old boy, the younger brother of case 1, presented with bilateral deafness, renal tubular dysfunction, failure to thrive, and mild cholestasis. He had the same mutations that were identified in his brother’s *VPS33B*. Case 3: A 24-year-old man born to nonconsanguineous Japanese parents was suspected to have progressive familial intrahepatic cholestasis 1 (PFIC1) in his childhood on the basis of low GGT cholestasis, renal tubular dysfunction, sensory deafness, mental retardation, and persistent itching. A liver biopsy performed at the age of 16 years showed findings that were consistent with PFIC1. He developed anemia owing to intraperitoneal hemorrhage from a peripheral intrahepatic artery the day after the biopsy, and transcatheter arterial embolization was required. ARCS2 was diagnosed using targeted NGS, which identified novel compound heterozygous mutations in *VIPAS39*.

**Conclusions:**

The first Japanese cases of ARCS1 and ARCS2 diagnosed using genetic tests were reported in this study. These cases are milder than those previously reported. For patients with ARCS, invasive procedures should be performed with meticulous care to prevent bleeding.

## Background

Arthrogryposis, renal dysfunction, and cholestasis syndrome (ARCS), a rare autosomal recessive disorder, is characterized by hypotonia-related arthrogryposis, renal tubular dysfunction, and neonatal cholestatic jaundice [1–3]. Additional clinical presentations of ARCS include ichthyosis, central nervous system anomalies, sensorineural deafness, failure to thrive, and platelet dysfunction. The prognosis of patients with ARCS is poor as most patients reportedly die of persistent infections and bleeding by the age of 1 year [2, 4]. Patients with ARCS have been reported to experience severe hemorrhaging after invasive procedures such as liver or kidney biopsy [2]. The prevalence of ARCS is known to be <1 in a million in Western countries, and only a few cases of ARCS have been reported worldwide [5, 6]. As ARCS is a rare disease with various clinical manifestations, its incidence may be underestimated.

Germline mutations in *VPS33B* and *VIPAS39* cause ARCS1 (OMIM: 208085) and ARCS2 (OMIM: 613404), respectively [1, 3]. As per the Global Variome (August 2021), the Leiden Open source Variation Database for ARC had compiled a total of 239 and 35 unique public variants in *VPS33B* and *VIPAS39*, respectively.

Here, we report three Japanese patients with a history of neonatal cholestasis in their infancy and who were diagnosed with ARCS1 and ARCS2 with compound heterozygous mutations in *VPS33B* and *VIPAS39*, respectively. Although these patients showed mild phenotypes with prolonged survival compared with the previously reported cases of ARCS, they presented with persistent bleeding after invasive procedures despite a normal platelet count and morphology in peripheral blood smears as well as normal prothrombin time-international normalized ratio (PT-INR).

## Case presentation

### Case 1

Case 1 is a 6-year-old boy born to nonconsanguineous Japanese parents. He was born at a gestational age of 39 weeks and 6 days and weighed 3,462 g at birth. The pregnancy and family history were unremarkable.

He was hospitalized for pneumonia at 22 days after birth, and jaundice and pale-colored stools were noted. Jaundice with a normal serum gamma-glutamyl transferase (GGT) level (28 U/L) was attenuated with ursodeoxycholic acid administration. He had proteinuria, bilateral nerve deafness, motor delay, and failure to thrive. When he was referred to our hospital at the age of 7 months, his liver function test and serum bilirubin level were normal; however, he suffered from retractable pruritus with a high serum total bile acid level (115.0 μmol/L). The patient’s growth has been unremarkable without any significant infections. When cochlear implantation was performed for deafness at the age of 2 years, he presented with persistent bleeding despite a normal platelet count (584 x 10^3^/μL), platelet morphology, and PT-INR (0.85). Moreover, he required hematoma removal after the operation.

**Fig. 1.**
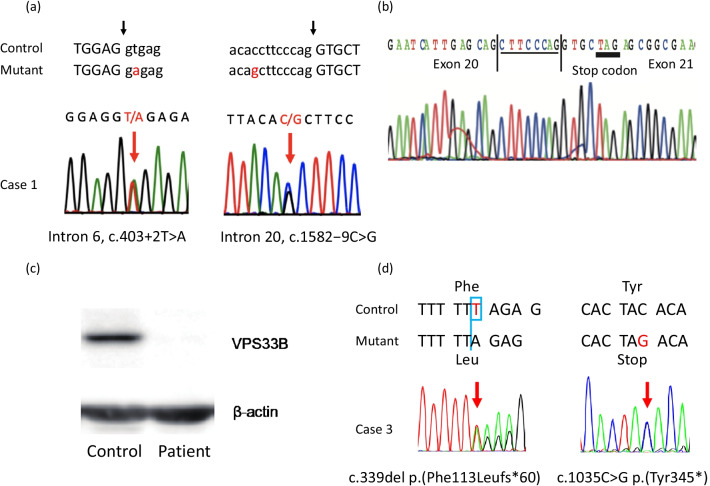
Genetic analysis of cases 1 and 3. **a** Genomic DNA sequences of *VPS33B*. Compound heterozygous mutations in *VPS33B* [NM_018668.4:c.403+2T*>*A in intron 6 and c.1582-9C>G in intron 20] were identified. The black arrows indicate exon–intron boundary. **b** cDNA sequence of *VPS33B*. Mutant mRNA derived from lymphocytes showed an insertion of an 8-bp intronic sequence after exon 20, resulting in a premature stop codon. **c** Western blot analysis of VPS33B expression. VPS33B expression was not detected in patients’ lymphocytes using the VPS33B antibody (sc-398322, Santa Cruz), which recognizes amino acids 271–570 of VPS33B. **d** Genomic DNA sequences of *VIPAS39*. Compound heterozygous mutations in *VIPAS39* {[NM_001193314.1:c.339del p.(Phe113Leufs*60)] and [NM_001193314.1:c.1035C>G p.(Tyr345*)]} were identified.

Although he did not present with arthrogryposis, he was suspected to have ARCS based on his other clinical symptoms. Using targeted next-generation sequencing (NGS) [7], compound heterozygous mutations were detected in *VPS33B* [NM_018668.4:c.403+2T*>*A in intron 6 and c.1582-9C>G in intron 20], which suggested that he had ARCS1 (Fig 1a). While c.403+2T*>*A is a well-known splice-site mutation [4, 8], c.1582-9C>G is a novel mutation located near the splicing acceptor site at the −9 position of intron 20. To investigate whether this novel mutation generates aberrant splicing products, cDNA generated from mRNA, which was extracted from whole blood, was sequenced. It was found that c.1582-9C>G mutation disrupts splicing, which generates cDNA with an abnormal insertion of an 8-bp intronic sequence after exon 20 and results in a premature stop codon (Fig 1b). Western blotting was then performed to confirm the effects of the mutations on protein expression. The VPS33B protein was not detected in the leukocytes of the patient using the VPS33B antibody (sc-398322, Santa Cruz), which recognizes amino acids 271–570 of VPS33B (Fig 1c).

**Fig. 2.**
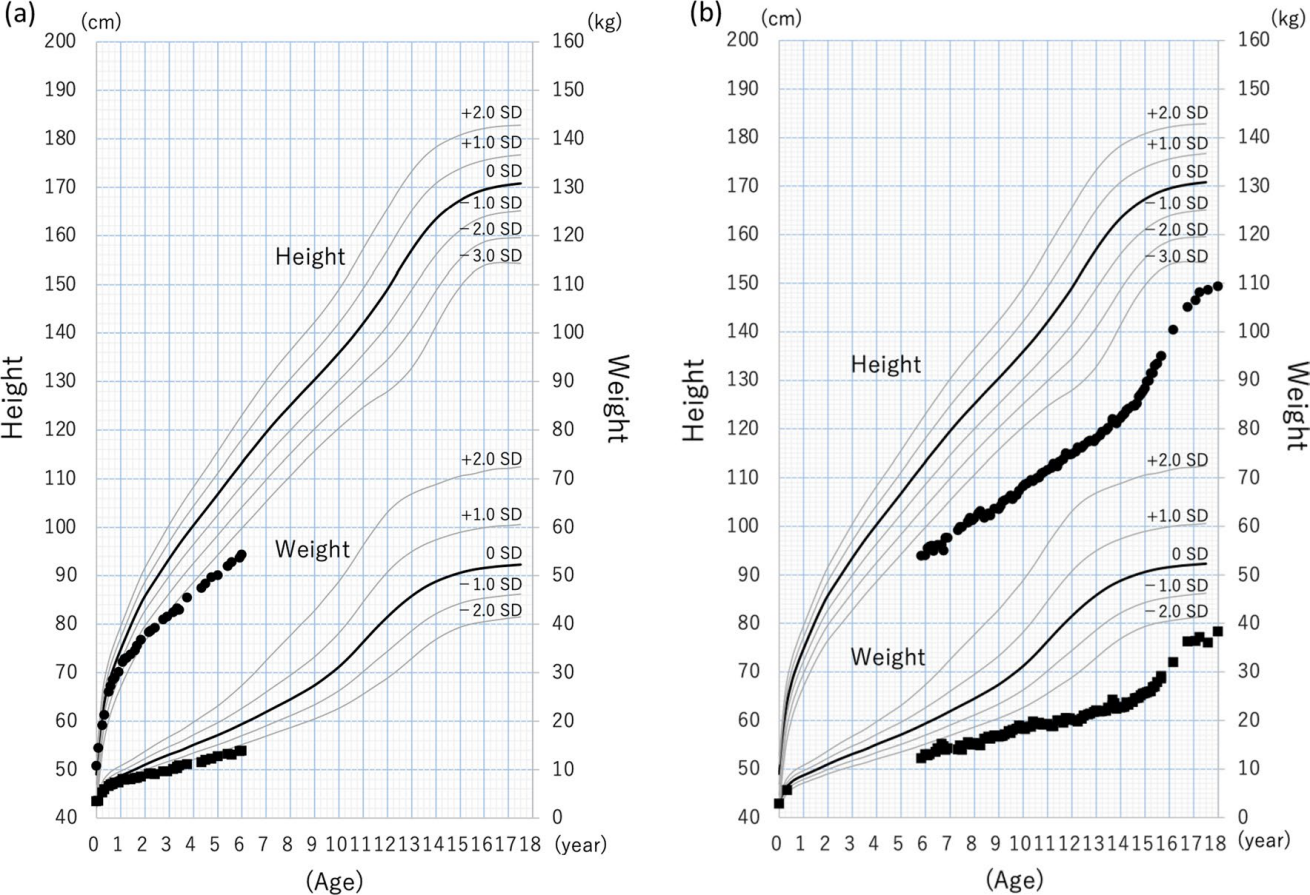
Growth charts of cases 1 and 3. Height and weight growth charts of cases 1 (**a**) and 3 (**b**) along with SD score lines for a standard Japanese male

At the age of 6 years, his height (standard deviation [SD]: −4.30) and weight (SD: −2.87) are below average; however, he shows gradual growth (Fig 2a). His psychomotor development has been slower than average and is developing gradually. He held up his own head at 6 months, sat up straight at 14 months, and stood with support at 21 months. He learned to use two-word sentences in sign languages at the age of 21 months. His developmental quotient (DQ) using the Kyoto Scale of Psychological Development 2001 [9] at the age of 3 years and 8 months was 38, corresponding to a developmental age of 1 year and 5 months. He started to walk on his own at the age of 5 years and 5 months and is currently attending a special support education school. His transaminase levels have been approximately 100 U/ml, and his serum GGT level has not shown any significant increase. Although his serum bilirubin levels have been normal, his serum total bile acid level is elevated and he still suffers from refractory pruritus.

### Case 2

Case 2, who is currently 9 months old, is the younger brother of case 1. He was born at a gestational age of 37 weeks and 3 days, with a birth weight of 3,090 g, after an uncomplicated pregnancy. He also did not have arthrogryposis; however, he was suspected to have ARCS1 as he presented with bilateral deafness, renal tubular dysfunction, failure to thrive, and mild cholestasis, similar to his elder brother. Sanger sequencing of his genomic DNA revealed the same mutations in *VPS33B*. Although his jaundice had ameliorated, he started to show signs of pruritus at the age of 6 months.

### Case 3

Case 3 is a 24-year-old man born to nonconsanguineous Japanese parents. He was born at a gestational age of 41 weeks and 2 days, with a birth weight of 2,806 g. The pregnancy and family history were unremarkable.

Failure to thrive and psychomotor developmental delay has been observed in this patient since infancy. He held up his own head at 6 months, sat up straight at 8 months, and walked by himself at 48 months. He started experiencing pruritus at approximately 7 months of age. He had cholestasis with normal serum GGT levels, renal tubular dysfunction, and bilateral nerve deafness. He was suspected to have progressive familial intrahepatic cholestasis 1 (PFIC1) on the basis of the clinical course and was referred to our hospital at the age of 15 years. Blood tests during the first visit to our hospital revealed mildly elevated aspartate aminotransferase (63 U/L), alanine aminotransferase (58 U/L), and total bile acid (90.8 μmol/L) levels; however, his GGT level (21 U/L) was normal. Urinary β_2_ microglobulin level, which indicates renal tubular dysfunction, was also elevated (76,729 ng/mL).

Liver biopsy at the age of 16 years revealed mild inflammatory cell infiltration in the lobule and portal vein area, with mild portal or pericellular fibrosis as well as bile plug in the lobule, consistent with PFIC1. However, the day after the biopsy, he developed anemia owing to intraperitoneal hemorrhage despite a normal PT-INR (0.97), platelet count (176 x 10^3^/μL), and platelet morphology in peripheral blood smears. Arterial imaging revealed hemorrhage from a peripheral intrahepatic artery, and hemostasis via transcatheter arterial embolization was required.

We performed a targeted NGS panel that evaluated 61 genes for infantile cholestatic diseases and identified novel compound heterozygous mutations in *VIPAS39*. One allele contained a single nucleotide deletion mutation [NM_001193314.1:c.339del p.(Phe113Leufs*60)], which modified the 60^th^ codon to a stop codon. The other allele contained a nonsense mutation [NM_001193314.1:c.1035C>G p.(Tyr345*)] (Fig 1d). As both of these mutations were pathogenic [10], he was diagnosed with ARCS2.

At 17 years old, his DQ was 21, corresponding to a developmental age of 3 years and 10 months as per the Kyoto Scale of Psychological Development 2001. His transaminase levels have remained normal since the age of 16 years, and his GGT level has not increased. He does not have jaundice but is experiencing continuous pruritus. This patient is currently 24 years old, with a height of 151.2 cm (SD: −3.36) and a weight of 43.0 kg (SD: −3.38) (Fig 2b). He currently works in a workshop.

## Discussion and conclusions

Patients with ARCS present with various phenotypes. These include the three main features (arthrogryposis, renal dysfunction, and cholestasis), which are accompanied by many other systemic symptoms, such as recurrent febrile illnesses, ichthyosis, bleeding tendency, deafness, mental retardation, and central nervous system anomalies [2, 4, 6]. Most patients with ARCS have been reported to die by the age of 1 year owing to recurrent infections and bleeding [2, 4]. ARCS is referred to as low- or normal GGT cholestasis along with some types of PFIC and bile acid synthesis disorders [2, 11, 12]. It should be noted that it is sometimes difficult to distinguish patients with ARCS from those with PFIC1, which shares the symptoms of short stature, diarrhea, hepatosplenomegaly, renal tubular dysfunction, and hearing loss along with cholestasis, as in our case 3 [12].

ARCS1 and ARCS2 are caused by mutations in either *VPS33B* or *VIPAS39*; these encode VPS33B and VIPAR, respectively. VPS33B and VIPAR form a functional complex that interacts with RAB11A. RAB11A plays a role in the apical recycling pathway and is also associated with other steps in intracellular vesicle transport, which include trafficking from the early endosomes to the trans-Golgi network and secretion via recycling endosomes [3]. VPS33B and VIPAR are expressed in many organs of the body, including the kidneys, liver, heart, lungs, brain, skin, and skeletal muscles, [1, 3, 6, 13], which explain the multisystem symptoms shared by ARCS1 and ARCS2 [14].

Compared with most of the previous reports, none of our patients showed any signs of arthrogryposis, their symptoms were mild, and the older patients are still alive at the ages of 6 and 24 years. In recent years, as in our cases, there have been some reports of incomplete ARCS phenotype without arthrogryposis as well as of milder ARCS with prolonged survival [14–17]. The mutations of c.1225+5G>C and c.1726T>C in *VPS33B* are known to be associated with a milder ARCS phenotype [14, 17]. In case 1, c.403+2T*>*A and c.1582-9C>G mutations were detected in *VPS33B*. The former is already a well-known mutation, and patients with this mutation have been reported to die within the age of 1 year [4]. The latter is a novel mutation that might be responsible for the mild phenotype. In case 3, novel pathogenic *VIPAS39* mutations were found (c.339del p.(Phe113Leufs*60), c.1035C>G p.(Tyr345*)). There are only few reports on ARCS2, and it is necessary to accumulate cases and investigate the genotype–phenotype correlation of ARCS2 in the future.

Patients with ARCS have been reported to have severe hemorrhaging, and it can be sometimes life-threatening or even fatal after liver or kidney biopsy [2, 18]. This abnormality is attributed to the dysfunction of platelets rather than the decreased count in the peripheral blood. Platelets from patients with ARCS show abnormal morphology; the platelets are large and pale due to the lack of normal granulation, and electron microscope images show a lack of α granules. In one report, abnormal platelet morphology in peripheral blood smears was detected in all cases [19], whereas in another study, abnormal platelet morphology was found in approximately 11% of cases only [2]. However, bleeding episodes were observed even in cases without morphological abnormalities. In our cases, although patients did not show an abnormal morphology in peripheral blood smear specimens, severe bleeding still occurred. In cases with suspected ARCS, invasive procedures (including biopsy) for diagnosis should be avoided as much as possible. When necessary, such procedures should be performed with meticulous care, even without an abnormal platelet count and morphology.

In conclusion, three cases of ARCS with novel mutations showing milder phenotypes with prolonged survival were reported in this study. It may be difficult to clinically distinguish the mild cases of ARCS from other cholestatic diseases with normal GGT (such as PFICs). However, if suspected, it is necessary that ARCS be diagnosed using genetic testing rather than liver biopsy, which is associated with a risk of fatal bleeding.

## Data Availability

The clinical data and images of all three patients are available from the corresponding author upon reasonable request.

## Abbreviations

ARCS: Arthrogryposis, renal dysfunction, and cholestasis syndrome
GGT: Gamma-glutamyl transferase
PT-INR: Prothrombin time-international normalized ratio
NGS: Next-generation sequencing
SD: Standard deviation
PFIC: Progressive familial intrahepatic cholestasis
DQ: Developmental quotient
